# Radiation induces changes in toll-like receptors of the uterine cervix of the rat

**DOI:** 10.1371/journal.pone.0215250

**Published:** 2019-04-18

**Authors:** Marie Francoise Mukanyangezi, Lucie Podmolíková, Wurood Al Hydad, Gunnar Tobin, Daniel Giglio

**Affiliations:** 1 Department of Pharmacology, Institute of Neuroscience and Physiology, Sahlgrenska Academy at the University of Gothenburg, Gothenburg, Sweden; 2 Department of Medical Biochemistry, Faculty of Medicine, Charles University, Hradec Králové, Czech Republic; 3 Department of Oncology, Institute of Clinical Sciences, Sahlgrenska Academy at the University of Gothenburg, Gothenburg, Sweden; Dasman Diabetes Institute, KUWAIT

## Abstract

Radiotherapy is an important therapeutic approach against cervical cancer but associated with adverse effects including vaginal fibrosis and dyspareunia. We here assessed the immunological and oxidative responses to cervical irradiation in an animal model for radiation-induced cervicitis. Rats were sedated and either exposed to 20 Gy of ionising radiation given by a linear accelerator or only sedated (controls) and euthanized 1–14 days later. The expressions of toll-like receptors (TLRs) and coupled intracellular pathways in the cervix were assessed with immunohistofluorescence and western blot. Expression of cytokines were analysed with the Bio-Plex Suspension Array System (Bio-Rad). We showed that TLRs 2–9 were expressed in the rat cervix and cervical irradiation induced up-regulation of TLR5, TRIF and NF-κB. In the irradiated cervical epithelium, TLR5 and TRIF were increased in concert with an up-regulation of oxidative stress (8-OHdG) and antioxidant enzymes (SOD-1 and catalase). G-CSF, M-CSF, IL-10, IL- 17A, IL-18 and RANTES expressions in the cervix decreased two weeks after cervical irradiation. In conclusion, the rat uterine cervix expresses the TLRs 2–9. Cervical irradiation induces immunological changes and oxidative stress, which could have importance in the development of adverse effects to radiotherapy.

## Introduction

Cervical cancer is the fourth most common cancer form among women [1]. While earlier stages are treated with surgery, advanced stages of cervical cancer are normally treated with radiotherapy alone or in combination with chemotherapy. The understanding on what happens in the normal tissue surrounding the tumour of the cervix upon exposure of radiation is at present lacking. Radiotherapy may cause vaginal mucosal atrophy, elastosis, fibrosis and vaginal stenosis and as a consequence dyspareunia [2, 3]. While the immune system has been studied in other irradiated tissues such as the colon [4], the lung [5] and the urinary bladder [6], the immune system of the irradiated cervicovaginal tract has not been studied. Pattern recognition receptors (PRRs) are part of the innate immune system and respond to pathogen-associated molecular patterns (PAMPs), which are constituted of microbial pathogens, and damage-associated molecular patterns (DAMPs). Toll-like receptors (TLRs) are an important group of receptors among PRRs. TLRs are expressed in different tissues including the cervix and respond to the exposure of bacteria and viruses thereby activating an immunological response [7]. Studies show that the cervix of the rabbit expresses TLRs 2, 3, 4, 5, 6, 8 and 10 [8]. Activation of TLRs induces intracellular activation of myeloid differentiation primary response 88 (MyD88) and TIR-domain-containing adapter-inducing interferon-β (TRIF) and via transcriptional factors (e.g. NFκB) may induce cytokine release [9].

It is tempting to assume that radiation causes the release of antigens from tumour cells and surrounding tissue activating DAMPs such as TLRs [10]. Numerous studies are undergoing to test TLR agonists in the therapy against cancer [11]. The TLR 7/8 agonist imiquimod is used in the therapy of HPV-related intraepithelial neoplasia including of the cervix and vagina [12]. The understanding of the immunological events following irradiation of the cervix could mean discovery of potential targets in the therapy of cervical cancer as well as finding therapeutic approaches to prevent the development of adverse effects to radiotherapy such as fibrosis. Activation of certain immunological pathways may lead to later development of fibrosis as has been demonstrated for radiation-induced dermal fibrosis, colonic fibrosis and pulmonary fibrosis [4, 13, 14]. In an animal model for radiation cystitis, we observed that radiation may suppress TLR4 and immunoregulatory pathways and affect the antioxidative system of the urinary bladder [6, 15]. Therefore, we presently assessed in an animal model how the innate immune system and antioxidative system of the cervix respond to the exposure of high-dose ionising radiation in the early phase following cervical irradiation. The radiation dose of 20 Gy used and the time window of two weeks assessed in the present model were chosen based on our findings in our rat model of radiation cystitis [6].

## Materials and methods

### Uterine cervix irradiation

Sprague-Dawley female rats (200–250 g; n = 49; CD IGS rat, Charles River, Germany) had free access to food and water. On the day of cervix irradiation, rats were sedated with pentobarbitone and medetomidine (50 mg/kg *im* and 10 μG/kg *ip*) and placed in the supine position with the legs placed on the abdomen (to keep the legs away from the radiation field). The uterine cervix was then radiated with one fraction of 20 Gy given by a linear accelerator (Varian Medical Systems Inc., Palo Alto; 6 MV). Radiation was given in two side-fields to minimize the exposure of the spinal cord. One dose of 20 Gy corresponds to an equivalent dose of 92 Gy in 2 Gy fractions (assuming α/β = 3). Control rats were only sedated. Rats were euthanized 1, 3, 7 and 14 days after uterine cervix irradiation by an overdose of pentobarbitone.

### Microscopical examination of cervical specimens

The thickness of the epithelium and the submucosa and the degree of infiltration of granulocytes were estimated in a blindly manner. The average thickness of the epithelium and the submucosa was calculated from nine randomly positions of each cervical specimen. The number of granulocytes in the cervical submucosa was graded 0 to 5 (0 = solitary granulocytes in the submucosa, 3 = 2–3 zones in the submucosa with high concentration of granulocytes, 5 = predominant part of the submucosa with high concentration of granulocytes).

### Immunohistofluorescence

Sections of cervical specimens (6 μM) were deparaffinised in xylene and rehydrated in decreasing concentrations of ethanol (99–95%) and tap water and washed in phosphate-buffered saline (PBS; Life technologies Ltd, Paisley, UK). The sections were boiled for 30 min in citrate buffer (pH 7.5) for antigen retrieval and incubated for two hours with CuSO_4_ solution to quench autofluorescence (Sigma-Aldrich, St. Louis, MO, USA). Unspecific protein binding was blocked by incubating sections for one hour in 5% goat serum (diluted in PBS). Incubation with the primary antibody (diluted in 1% goat serum in PBS; see S1 Table) occurred overnight at 4°C. The next day the sections were washed in PBS and incubated with the secondary antibody (1:250; 1% goat serum in PBS; see S1 Table) for one hour. After washes in PBS, sections were dehydrated in increasing concentration of ethanol (95–99%). Prolong Gold Antifade Reagent with DAPI (Life technologies) was applied on the sections and cover glasses were then mounted. The average pixel intensity of antigen staining in one representative area of epithelium, submucosa, smooth muscle and submucosal blood vessel wall in every cervix specimen was measured with Photoshop (version 12.0.4).

### Immunohistochemistry for CD3+ lymphocytes

To detect CD3+ lymphocytes in the cervical tissue, HRP/DAB immunohistochemistry was performed (Abcam; Mouse and Rabbit Specific HRP/DAB (ABC) Detection IHC Kit; ab64264). In brief, after deparaffinization and hydration (see above), the sections were blocked with hydrogen peroxide. Antigen retrieval was then performed as above and protein block was applied on the sections. Incubation with the primary antibody against CD3 occurred overnight at 4°C. Sections were the next day incubated with biotinylated goat anti-polyvalent followed by streptavidin peroxidase followed by applying DAB chromogen mixed with DAB substrate on the sections for 5 min. After counterstaining with Mayer’s hematoxylin (Histolab Products AB, Göteborg, Sweden) the sections were washed in tap water and dehydrated in increasing concentrations of ethanol and xylene. Sections were covered with Pertex mounting medium (Histolab Products AB) and cover slips. The average number of CD3+ lymphocytes present in the epithelium and submucosa per three vision fields (x100) was counted in the cervical specimens (n = 7).

### Protein estimations and western blot analysis

Cervix specimens were homogenized in homogenization buffer containing 0.1% phosphatase inhibitor cocktail 2 (Sigma-Aldrich) and 0.5% protease inhibitor cocktail (Sigma-Aldrich), centrifuged and the supernatants were recovered. The Pierce BCA Protein Assay Kit (Thermo Scientific, Rockford, IL, USA) was used to determine the concentrations of the protein samples. The protein samples were mixed with NuPAGE LDS Sample Buffer (Life Technologies) and NuPAGE Reducing Agent (Life Technologies) and heated for 10 min at 70°C. Proteins were separated by electrophoresis on NuPAGE 4–12% Bis-Tris gels (Life Technologies) in MOPS buffer (Life technologies Ltd) followed by transferring the proteins onto nitrocellulose membranes (Life Technologies) for one hour at 30 V. The membranes were washed in tris-buffered saline with 0.3% Tween 20 (TBS-T; Sigma-Aldrich) and blocked for one hour with 5% non-fat milk in TBS-T. After washes in TBS-T, incubation with the primary antibody (in TBS-T containing 3% goat serum; see S1 Table) occurred overnight at 4°C. On the next day, after washes in TBS-T, the membranes were incubated with the secondary antibody (in TBS-T containing 5% non-fat milk; see S1 Table) for one hour. Signals of binding of the antibodies were developed with Amersham ECL Plus Western Blotting Detection Reagent (GE Healthcare, Little Chalfont, UK) and visualized with the Fujifilm Image Reader LAS-1000 Pro v.2.6 (Stockholm, Sweden). Quantification of pixel intensity was made with the Fujifilm Multi Gauge v3.0 software (Stockholm, Sweden). Membranes were washed in TBS-T, stripped in Restore Western Blot Stripping Buffer (Thermo Fisher Scientific, Rockford, IL, USA) and washed again in TBS-T. The membranes were then blocked for 60 min in TBS-T containing 5% non-fat milk and incubated then with a new primary antibody followed by the protocol as above.

### Cytokine analysis from cervical specimens

Cytokines were analysed from homogenized cervical tissue with the Bio-Plex Pro Assays according to the instructions of the manufacturer (Bio-Rad Laboratories Inc, Irvine, CA, USA). Erythropoietin (EPO), granulocyte colony-stimulating factor (G-CSF), granulocyte-macrophage (GM)-CSF, Gro/KC (chemokine (C-X-C motif) ligand 1; CXCL1), interferon gamma (IFN-γ), interleukin (IL)-1α, IL-1β, IL-2, IL-4, IL-5, IL-6, IL-7, IL-10, IL-12p70, IL-13, IL-17A, IL-18, macrophage (M)-CSF, monocyte chemotactic protein 1 (MCP-1), macrophage inflammatory protein (MIP)-1α (CCL3), MIP-3α, regulated on activated, normal T-cell expressed and secreted (RANTES), tumour necrosis factor alpha (TNF-α) and vascular endothelial growth factor (VEGF) were analysed.

### Statistical analysis

Statistical significance for unpaired data was determined with the Mann-Whitney test. The Kruskal-Wallis test was used when multiple comparisons were made followed by the Dunn’s multiple comparison test. Linear regression was performed to assess the relationship between cervical TLR5 expression and the number of days following cervical irradiation and the coefficient of determination (r-squared) was determined. A p-value of 0.05 or less was considered as statistically significant. Data is presented in the form of mean±standard error of the mean if not stated otherwise. Graphs were generated and parameters computed using the GraphPad Prism program 7.0 (GraphPad Software, Inc., San Diego, US).

## Results

### Activation of the innate immune system and anti-oxidation

**Fig 1:**
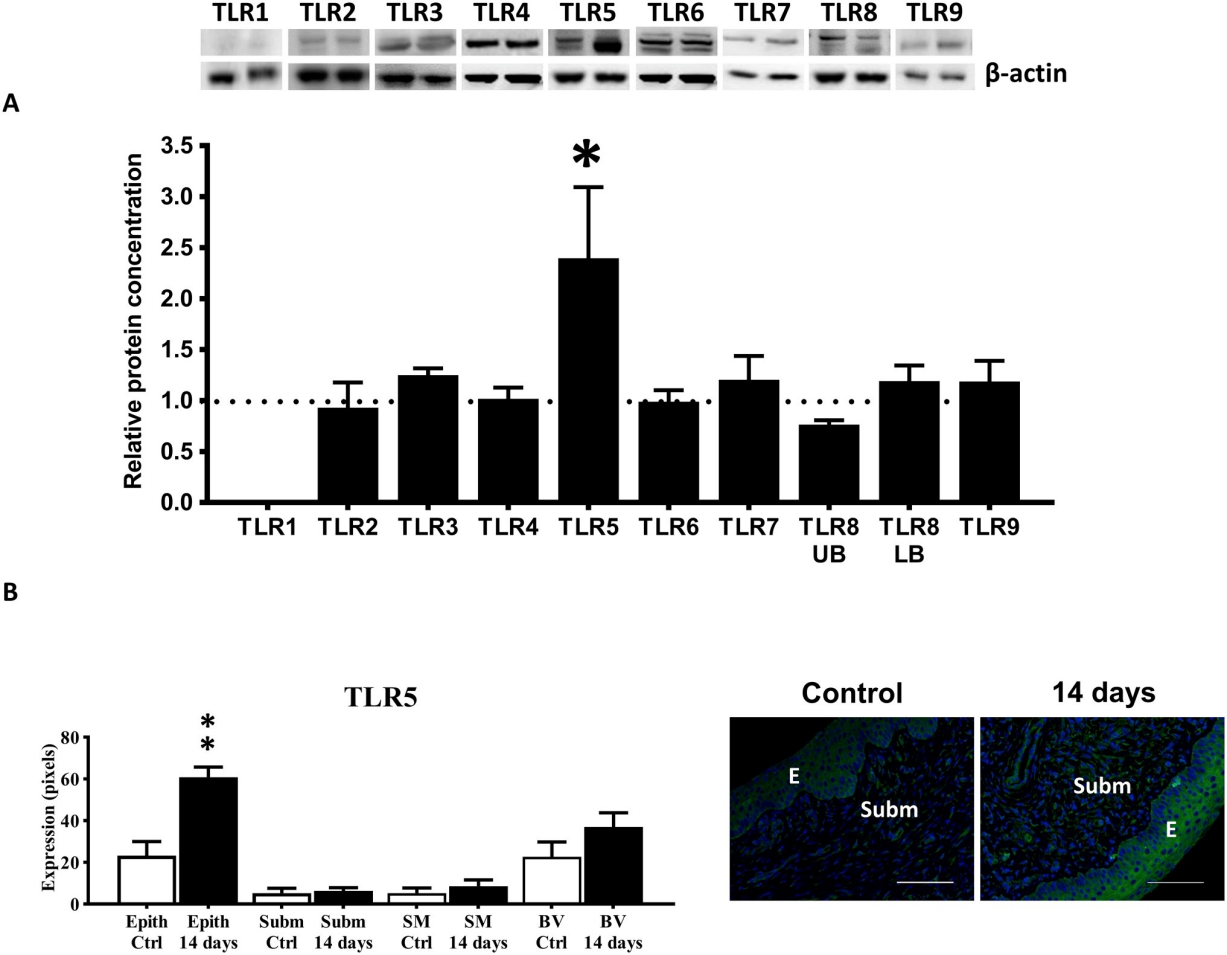
Expression of TLRs in control and irradiated cervical specimens. A) Western blot analysis of TLR1 (90 kDa), TLR2 (90 kDa), TLR3 (95 kDa), TLR4 (95 kDa), TLR5 (95 kDa), TLR6 (96 kDa), TLR7 (121 kDa), TLR8 upper band (95 kDa), TLR8 lower band (90 kDa) and TLR9 (100 kDa) in control and irradiated cervices at day 14 post-irradiation (n = 5–7; expressed as fraction of control cervix expression). B) TLR5 expression in different structures of the cervix (n = 7–8). Representative microphotographs immunostained for TLR5 (green) and DAPI-stained nuclei (blue) of a control and irradiated cervix to the right. *E* indicates epithelium and *Subm* indicates submucosa. Horizontal bars indicate 200 μm. * indicates p<0.05 between TLR5 expression in control and irradiated cervices at day 14 post-irradiation and ** indicates p<0.01 between TLR5 epithelial expression in control and irradiated cervices at day 14 post-irradiation. Vertical bars indicate S.E.M.

Histological examination of the cervix showed that irradiation induced oedema in the submucosa and the thickness of the submucosa increased time-dependently after irradiation (at days 7–14 post-irradiation; p<0.001; n = 5; S1 Fig). Granulocytes were particularly expressed in the submucosa and the granulocyte number was not significantly affected by cervical irradiation (S1 Fig). Neither was the number or distribution of CD3+ lymphocytes in the submucosa or epithelium affected by irradiation (S2 Fig). Western blot showed the expression of TLRs 2–9 but not the expression of TLR1 in control and irradiated cervical tissue. While TLR1 was not detected with western blot in cervical tissue with two antibodies tested (see Methods), TLR1 was expressed for example in the rat brain (10.6084/m9.figshare.7886066). An increase in cervical TLR5 expression in response to cervical irradiation occurred and the increase occurred time-dependently (p<0.05; n = 6; Fig 1a and S3 Fig). The increase in TLR5 occurred in the cervical epithelium (p<0.01; n = 7; Fig 1b). TLRs 2–9 were expressed in normal, non-irradiated, cervical specimens (S4 Fig).

**Fig 2:**
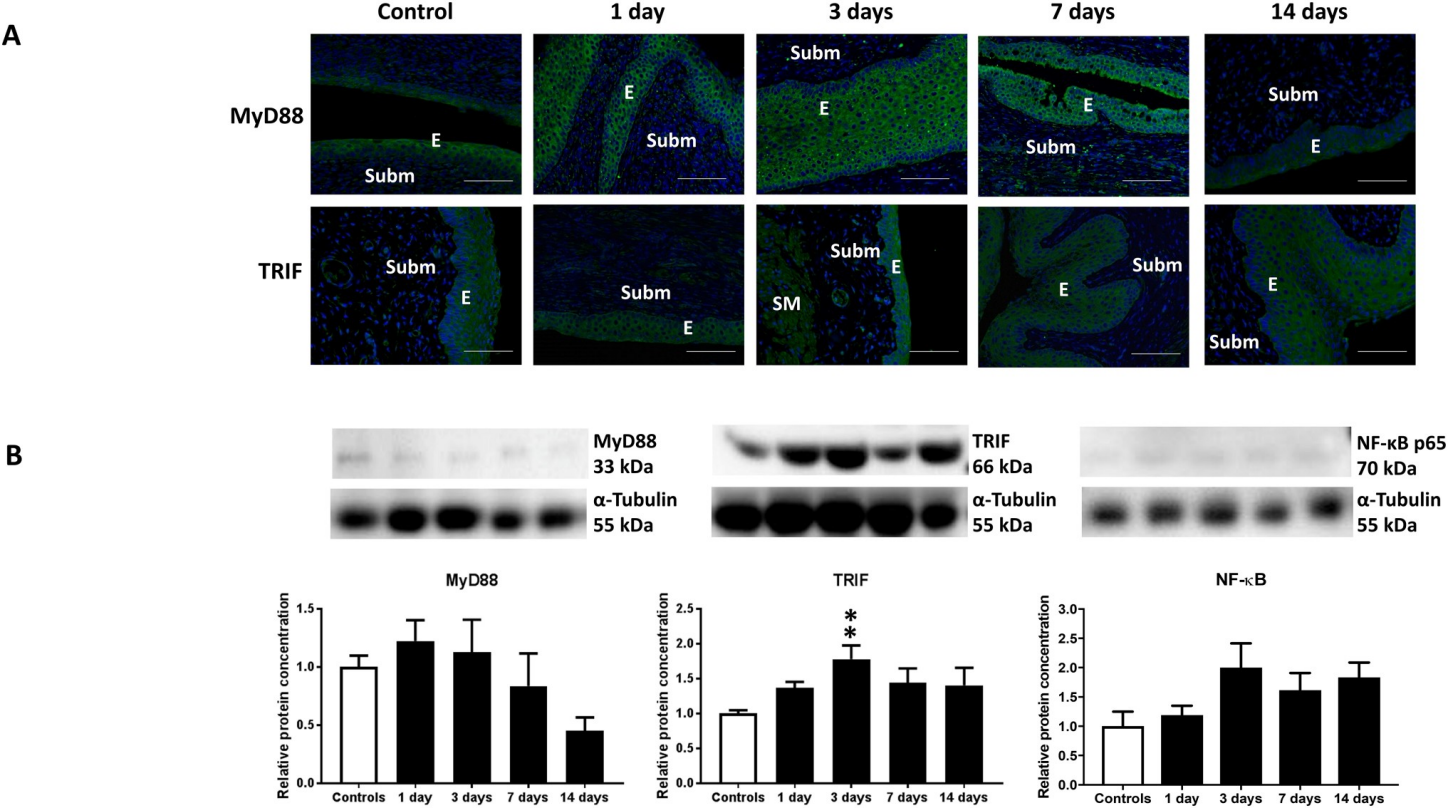
Activation of TLR-connected signaling pathways in response to cervical irradiation. A) Representative microphotographs immunostained for MyD88 (green), TRIF (green) and DAPI-stained nuclei (blue) of control and irradiated cervices (days 1–14 post-irradiation). *E* indicates epithelium, *Subm* indicates submucosa and *SM* indicates smooth muscle. Horizontal bars indicate 200 μm. B) Western blot analysis of MyD88, TRIF and NF-κB expression in control and irradiated cervices (n = 6). ** indicates p<0.01 between TRIF expression in control and irradiated cervices at day 3 post-irradiation. Vertical bars indicate S.E.M.

In general, the epithelium expressed a more intense staining signal for the TLRs than the underlying stroma. TLRs 2–9 were expressed in the epithelium. In the submucosa, TLR2, TLR4, TLR5, TLR6 and TLR9 were expressed. In the smooth muscle TLR2-9 except TLR8 were expressed. TLR4, TLR5, TLR6 and TLR7 were also expressed in blood vessels. MyD88 and TRIF were expressed in the cervical epithelium (Fig 2a). Western blot analysis showed that TLR downstream molecules TRIF and NF-κB were increased in the irradiated cervix, while MyD88 instead was decreased (Fig 2b).

**Fig 3:**
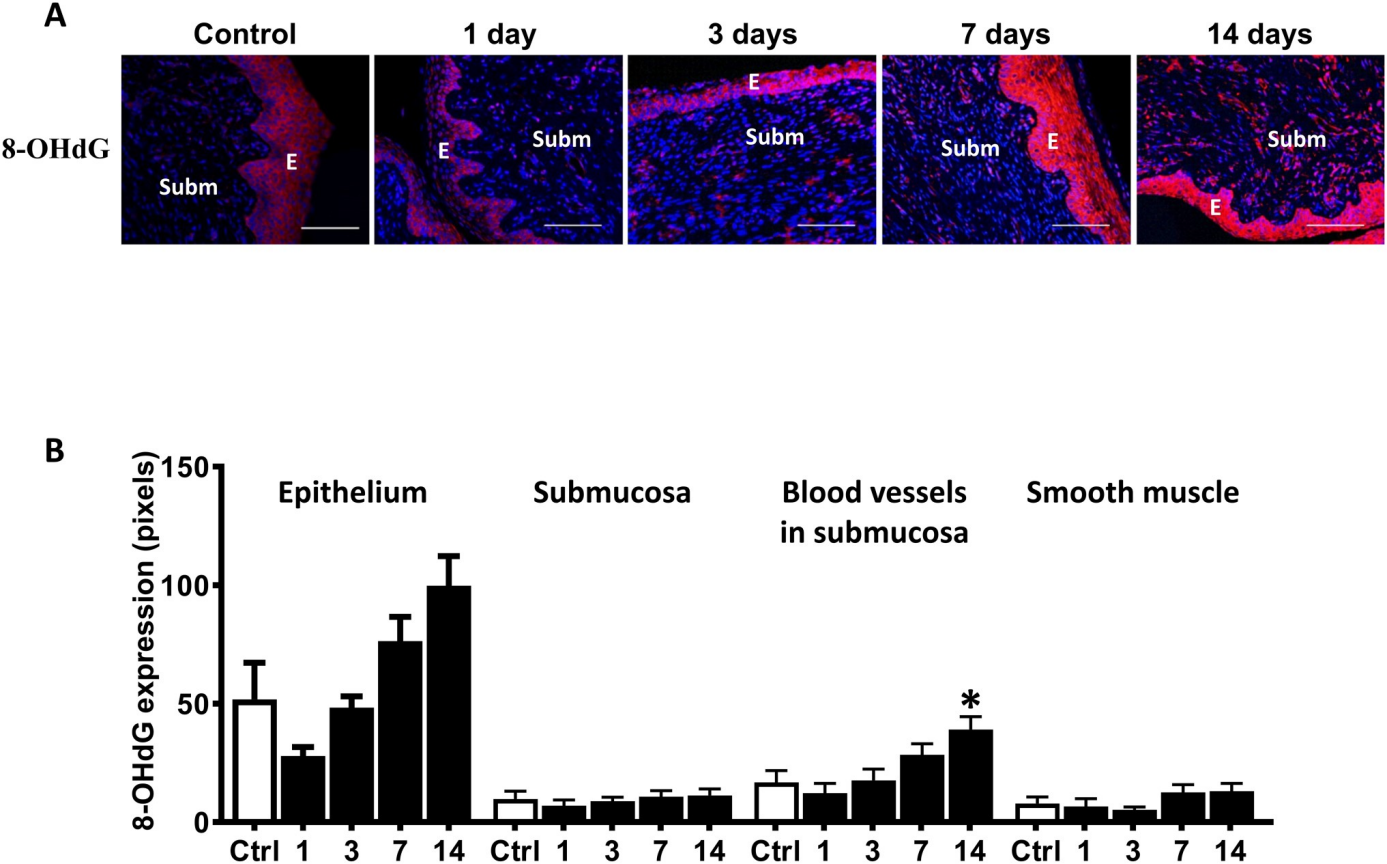
Increase in oxidative stress in response to cervical irradiation. A) Representative microphotographs immunostained for 8-OHdG (red) and DAPI-stained nuclei (blue) of control and irradiated cervices (at days 1–14 post-irradiation). B) 8-OHdG expression in different structures of the cervix (n = 5–10). *E* indicates epithelium and *Subm* indicates submucosa. Horizontal bars indicate 200 μm. * indicates p<0.05 between 8-OHdG expression in submucosal blood vessels of control and irradiated cervices at day 14 post-irradiation. Vertical bars indicate S.E.M.

**Fig 4:**
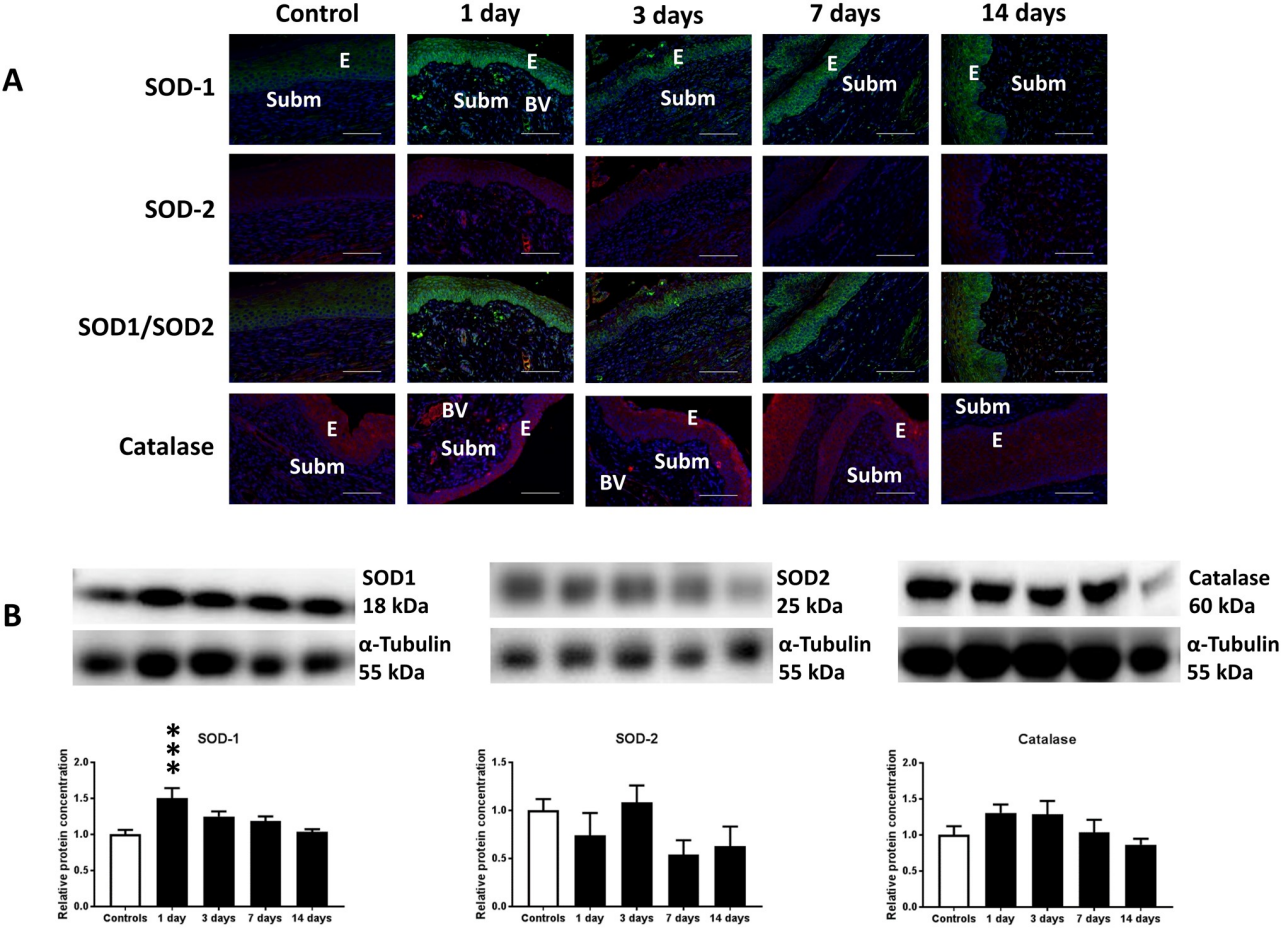
Increases in antioxidants in response to cervical irradiation. A) Representative microphotographs immunostained for SOD-1 (green), SOD-2 (red), merged SOD-1/SOD-2 (third row), catalase (red) and DAPI-stained nuclei (blue) of control and irradiated cervices (days 1–14 post-irradiation). *E* indicates epithelium, *Subm* indicates submucosa and *BV* indicates blood vessel. Horizontal bars indicate 200 μm. B) Western blot analysis of SOD-1, SOD-2 and catalase expression in control and irradiated cervices (days 1–14 post-irradiation; n = 6). ** indicates p<0.01 between SOD-1 expression in control and irradiated cervices at day 1 post-irradiation. Vertical bars indicate S.E.M.

8-OHdG, SOD-1, SOD-2 and catalase were expressed in the cervical epithelium and in blood vessels of the cervical submucosa (Figs 3a and 4a). Cervical irradiation induced up-regulation of 8-OHdG, SOD-1 and catalase in the epithelium and in submucosal blood vessels of the cervix (Figs 3a, 3b, 4a and 4b).

### Cytokine expressions after irradiation

G-CSF, M-CSF, IL-10, IL- 17A, IL-18 and RANTES expressions in the cervix decreased two weeks after cervical irradiation (p<0.001–0.05; n = 10–16; Tables 1 and 2). GM-CSF and MCP-1 tended to decrease but significance was not attained (p = 0.05 and p = 0.06, respectively; n = 16; Tables 1 and 2).

**Table 1:** Cytokine expressions in control and irradiated cervical specimens.

	EPO	G-CSF	M-CSF	GM-CSF	GRO/KC	IL-1α	IL-1β	IL-7	IL-10
**CONTROL**									
Median:	9.94	1.91	8.16	6.98	15.97	61.07	46.28	12.02	388.6
Mean:	17.43	2.116	9.33	6.883	30.94	126.1	46.23	21.79	502.6
SD:	20.43	1.107	4.15	1.87	35.27	137.2	33.29	30.01	263.0
Min-Max:	4.92–76.9	1.05–4.94	5.38–20.98	2.99–10.94	6.26–130.9	4.65–486.2	2.65–118.6	0.92–126.2	278.1–1069
N:	16	16	16	16	16	16	16	16	16
**IRRADIATED**									
Median:	8.29	1.05	5.88	5.83	13.56	44.61	29.275	6.5	278.14
Mean:	14.36	1.156	5.823	5.861	22.25	210.2	40.59	12.18	307.2
SD:	13.35	0.2465	1.731	1.44	23.00	410.3	36.84	15.17	112.4
Min-Max:	4.92–53.14	1.05–1.91	2.32–8.61	2.86–8.96	6.26–81.33	4.2–1331	2.65–134.1	0.92–58.22	278.1–713.5
N:	16	16	16	16	16	16	16	16	15
Fold change (decrease):	1.20	**1.82**	**1.39**	1.20	1.18	1.37	1.58	1.85	**1.40**
P-value:	0.7088	**0.0006**	**0.0025**	0.0523	0.7874	0.4619	0.5198	0.2029	**0.0019**

Cytokine concentrations (pg/ml) of cervical tissue homogenates of control and irradiated cervices day 14 post-irradiation. SD = standard deviation, N = number of samples/animals.

**Table 2:** Cytokine expressions in control and irradiated cervical specimens.

	IL-17A	IL-18	MCP-1	MIP-1α	RANTES	TNF-α	VEGF
**CONTROL**							
Median:	2.14	2.92	23.26	2.19	5.55	18.78	72.42
Mean:	2.73	4.92	24.14	2.81	7.86	20.37	158.2
SD:	1.67	4.08	14.25	1.39	6.69	9.55	198.8
Min-Max:	1.25–7.17	1.39–1185	4.14–56.21	1.85–6.39	1.51–20.51	6.23–36.54	21.12–799.6
N:	16	16	16	16	16	16	16
**IRRADIATED**							
Median:	1.25	1.39	14.18	1.85	1.51	20.55	35.93
Mean:	3.17	1.80	15.05	2.53	4.47	19.1	128.7
SD:	7.04	1.54	8.304	1.61	5.26	6.13	224.4
Min-Max:	1.25–29.53	1.39–7.56	4.14–31.88	1.85–7.66	1.51–18.43	9.38–28.54	8.5–850.4
N:	16	16	16	16	16	16	16
Fold change (decrease):	**1.71**	**2.10**	1.64	1.18	**3.68**	0.91	2.02
P-value:	**0.006**	**0.0051**	0.0574	0.218	**0.0447**	0.8451	0.1306
**Not detected**	IFN-γ, IL-2, IL-4, IL-5, IL-6, IL-12p70, IL-13, MIP-3α						

Cytokine concentrations (pg/ml) of cervical tissue homogenates of control and irradiated cervices day 14 post-irradiation. SD = standard deviation, N = number of samples/animals.

## Discussion

In our study we wondered how radiation affects the immunological system of the uterine cervix with focus on the expression of TLRs. The expression of TLRs (TLR1-9) in the female reproductive tract has been demonstrated in previous studies [7, 16–18]. It is also established that TLR stimulation may boost radiation-induced immune responses in cancer [19]. However, knowledge about how radiation affects the expression of TLRs in the normal cervical tissue is at present lacking. The most apparent finding of our study was that cervical irradiation induced increases of TLR5 in the epithelium concomitant with changes in cervical levels of TLR adaptor molecules MyD88, TRIF and NF-κB. The cervical epithelium expressed TLRs 2–9, which indicates that the cervical epithelium may respond to a high number of PAMPs and DAMPs. In the epithelium, TLR4, TLR6 and TLR9 were more expressed than TLR2, TLR3, TLR5, TLR7 and TLR8. Previous studies on cervical human epithelial cell lines showed the presence of all TLRs (1–9), where stimulation of TLR2, TLR3, TLR5 and TLR6 induced the release of cytokines [20]. The myometrium expresses TLR2, TLR3 and TLR5 and stimulation of these TLRs may induce the release of pro-inflammatory and pro-labour mediators [21–23].

TLRs (such as TLR2, TLR4 and TLR5) may use the MyD88/IRAK/NF-κB signal and the TRIF transduction pathways. Our data show that TRIF and NF-κB increased while MyD88 decreased in the irradiated cervix. The increase in TRIF appeared in the epithelium concomitant with the up-regulation of TLR5. Studies show that TLR5 may interact with the TRIF pathway in the gut epithelium [24]. TLR5 may mitigate radiation-induced damage in the irradiated head and neck area of the mouse [25]. TLR5 agonists may also reduce apoptosis after irradiation of the gut thereby improving tissue remodelling after rectal irradiation [26, 27]. The TLR5 agonist flagellin protects mice exposed to a lethal dose of whole-body γ-irradiation via MyD88 dependent pathways [28]. Radioprotective effects have also been shown to be generated via other TLRs such as TLR2, TLR3, TLR4 and TLR9 [29].

Our study showed that oxidative stress (reflected by 8-OHdG staining) increased successively till 14 days after cervical irradiation, while SOD-1 and catalase increased in the cervical tissue already 24 hours after cervical irradiation. Changes in oxidative stress and antioxidants occurred particularly in the epithelium and in the submucosal blood vessels of the cervix. The present findings are in line with previous reports showing that oxidative stress and anti-oxidative responses appear particularly in the urothelium and in submucosal blood vessels 28 days following irradiation of the rat urinary bladder [30]. Our data may indicate that oxidative stress induced by cervical irradiation activated TLR5 and TLR-connected downstream molecules TRIF and NF-κB thereby mitigating oxidative stress via the release of antioxidants SOD-1 and catalase in the cervical tissue.

Irradiation induced edema in the cervical submucosa, however, irradiation did not affect the distribution and number of granulocytes or lymphocytes in the cervix. The dominant effect on the cervical tissue 14 days following irradiation was instead an anti-inflammatory response reflected by a reduction in important pro-inflammatory cytokines and chemokines. IL-17 was decreased concomitant with a decrease in G-CSF, M-CSF and IL-10 levels in the cervix. While we only measured the level of cytokines on day 14 following cervical irradiation we do not know if the suppression of cytokines was preceded by an increase in cytokines at an earlier time point. However, the present findings are in line with studies showing that pro-inflammatory mediators are reduced 14 days following urinary bladder irradiation [6, 30]. IL-17 plays an important role in the female genital tract and may be released by Th17 cells and mucosal-associated invariant T (MAIT) cells [31, 32]. Our findings with a reduced IL-17 in response to cervical irradiation may indicate that radiation may lead to a weaker response to pathogens. An imbalance between Th1 and Th2 cytokines may also favour the development of adverse functional side effects to radiation and fibrosis. A number of studies have shown that Th17 cells and IL-17 play important roles in the development of radiation-induced functional disorders and fibrosis, *e*.*g*., radiation-induced proctitis [4], liver fibrosis [33, 34] and radiation-induced pulmonary fibrosis [35, 36]. Interestingly, IL-10 and IL-17 levels in bronchoalveolar lavage after pulmonary irradiation were shown to be inversely correlated to the severity of pulmonary fibrosis [35]. Speculatively, our findings of reduced levels of IL-10 and IL-17 may indicate that a pro-fibrotic micro-environment may have been developed in the cervix. The development of fibrosis seems, however, to take time since we could not either observe fibrosis in the rat cervical tissue 28 days after cervical irradiation (unpublished data).

The understanding of how the uterine cervical tissue responds to radiotherapy is at present scarce. We here assessed the different components in the innate immune system of the uterine cervix and our study gives insight in the early events following cervical irradiation. This could have importance for how to intervene to prevent the development of fibrosis in the female pelvic area. Whether targeting TLRs or other key elements in the innate immune system may alleviate adverse effects to radiotherapy should be assessed in future studies.

## Conclusions

The present study shows that the rat uterine cervix expresses the TLRs 2–9. By exposing the rat cervix to radiation, TLR5 was activated together with an up-regulation of oxidative stress and antioxidative responses concomitant with a reduction in important pro-inflammatory cytokines.

## Supporting information

S1 Fig Morphometry and granulocytes in control and irradiated cervices. (TIF)

S2 Fig CD3+ cells in in control and irradiated cervical specimens. (TIF)

S3 Fig TLR5 expression in control and irradiated cervical specimens. (TIF)

S4 Fig Expressions of TLRs 2–9 in control and irradiated cervical specimens. (TIF)

S1 Table Information on antibodies used for immunohistochemistry and western blot. (DOCX)
