# Supplementary figures and images for: Radiation induces changes in toll-like receptors of the uterine cervix of the rat
Source: PLoS One. 2019 Apr 18;14(4):e0215250. doi: 10.1371/journal.pone.0215250 (PMC6472742; doi:10.1371/journal.pone.0215250)

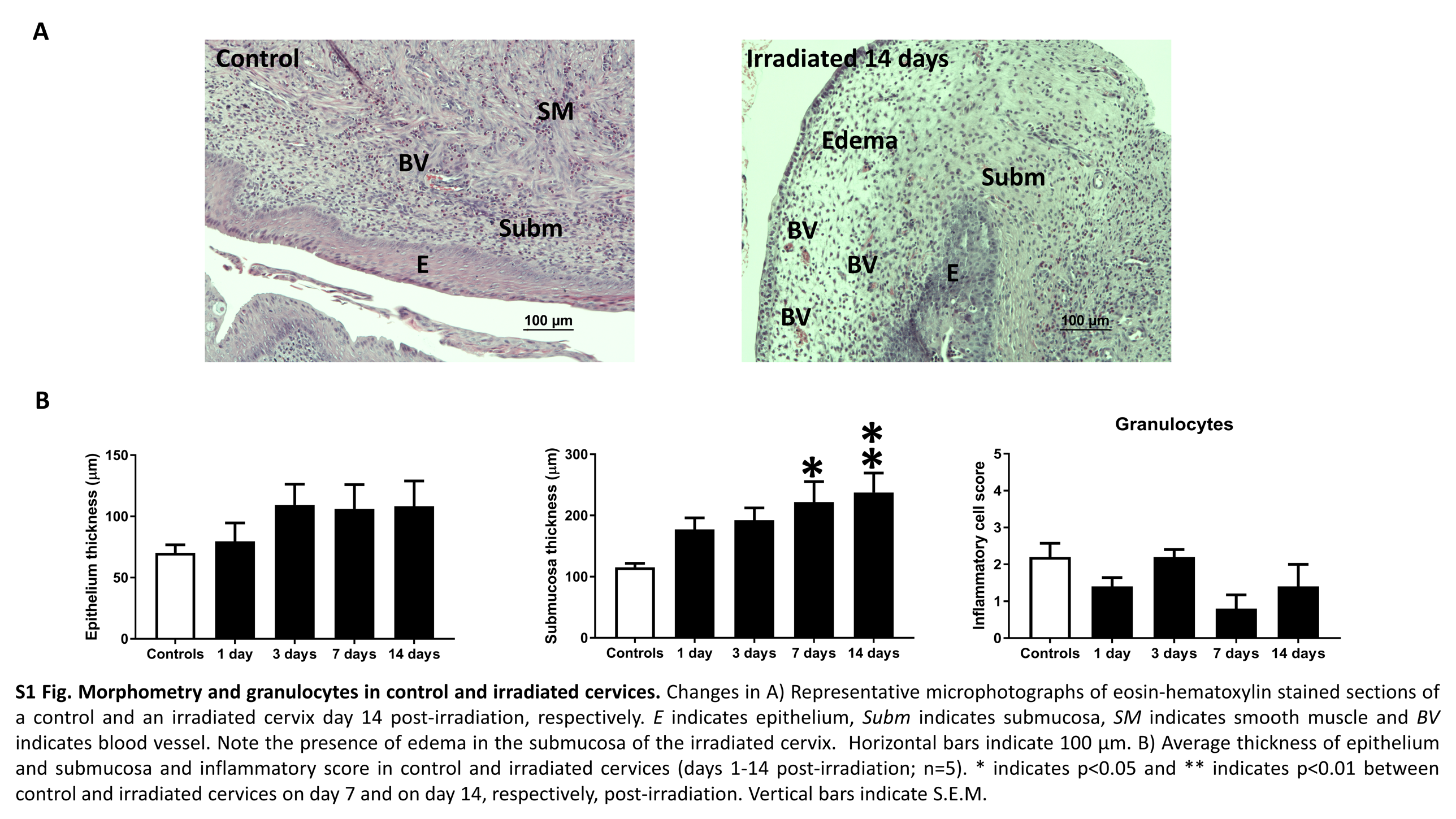

Supplement: S1 Fig — (TIF) [file pone.0215250.s001.tif]

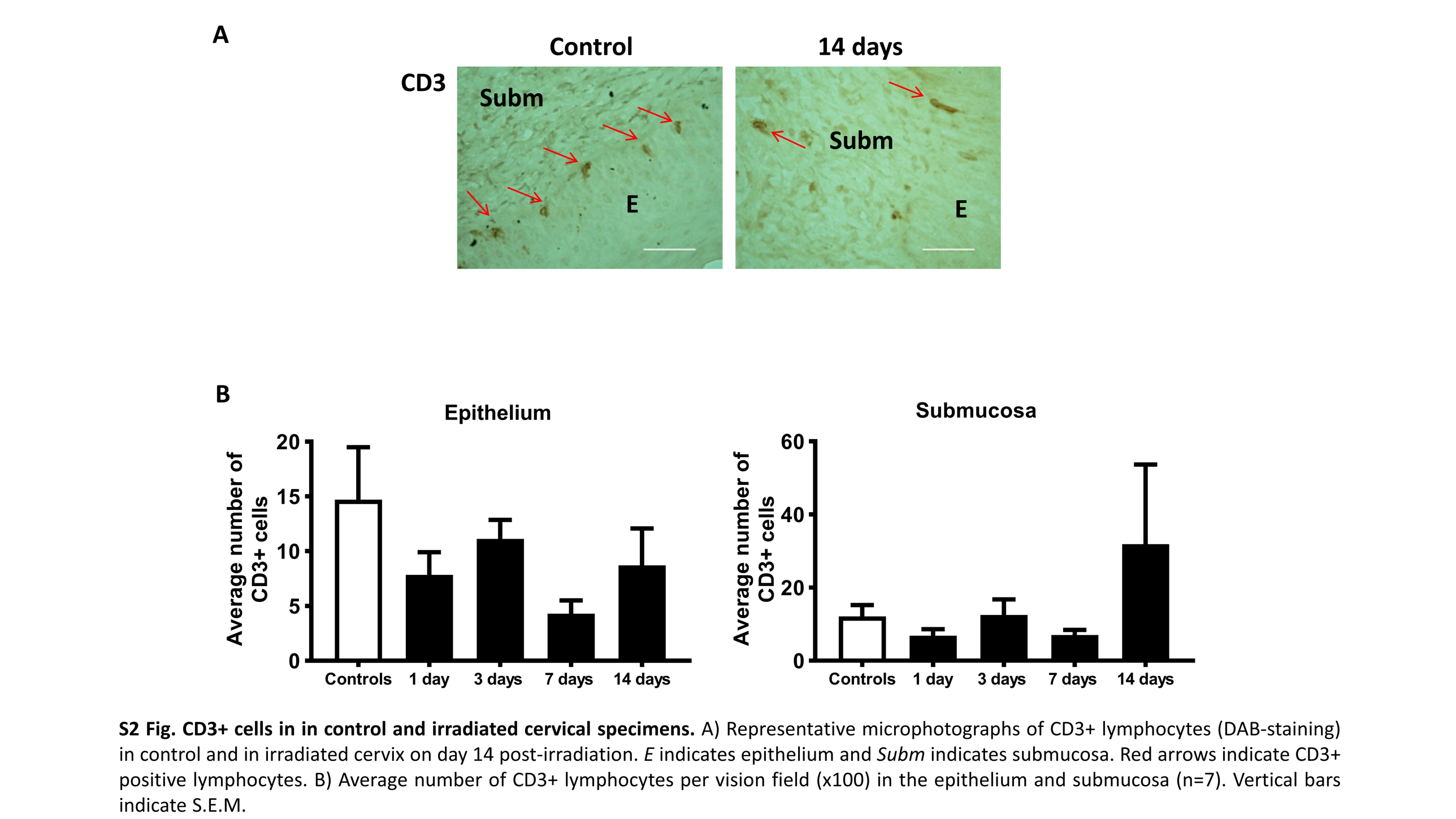

Supplement: S2 Fig — (TIF) [file pone.0215250.s002.tif]

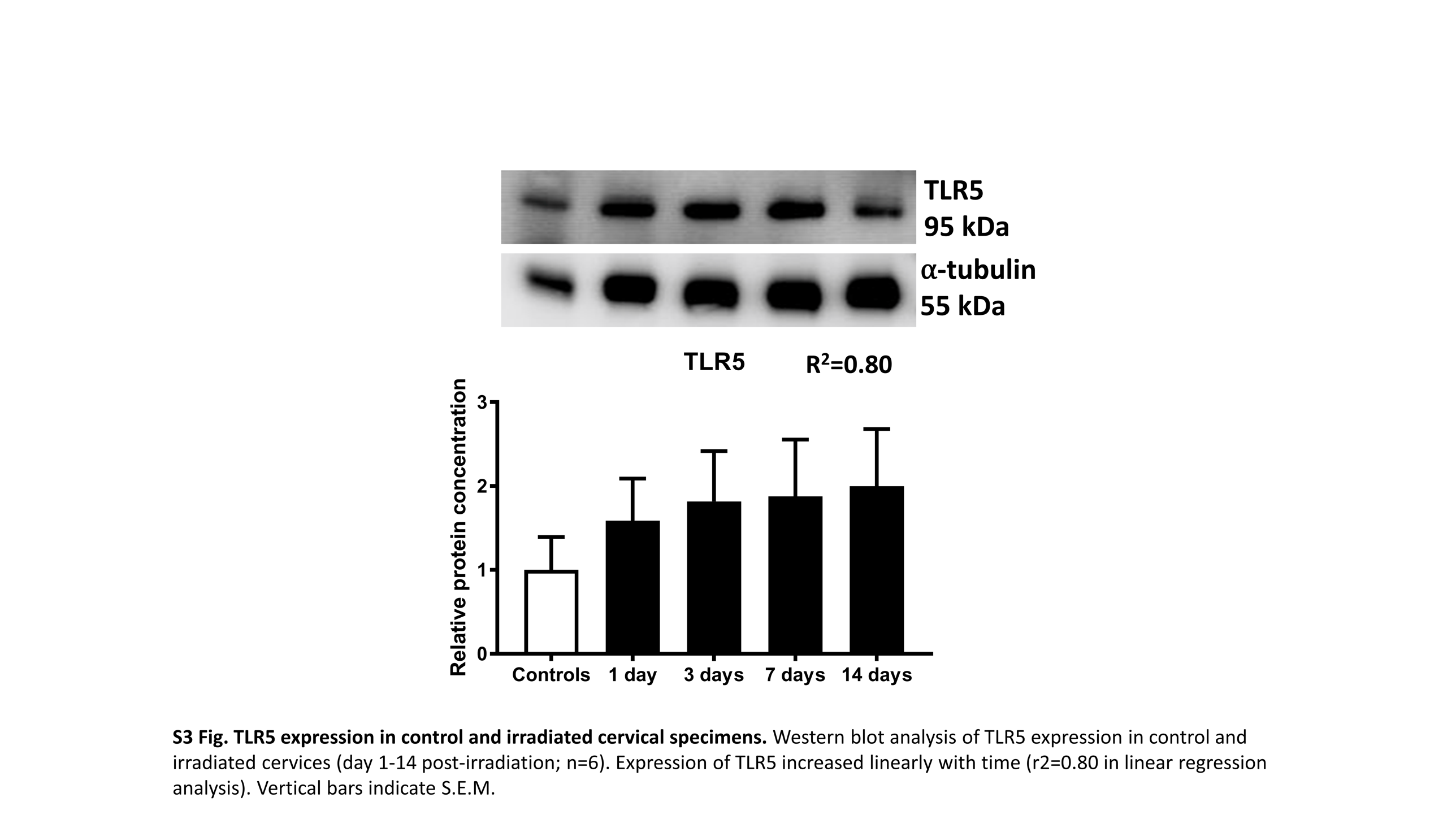

Supplement: S3 Fig — (TIF) [file pone.0215250.s003.tif]

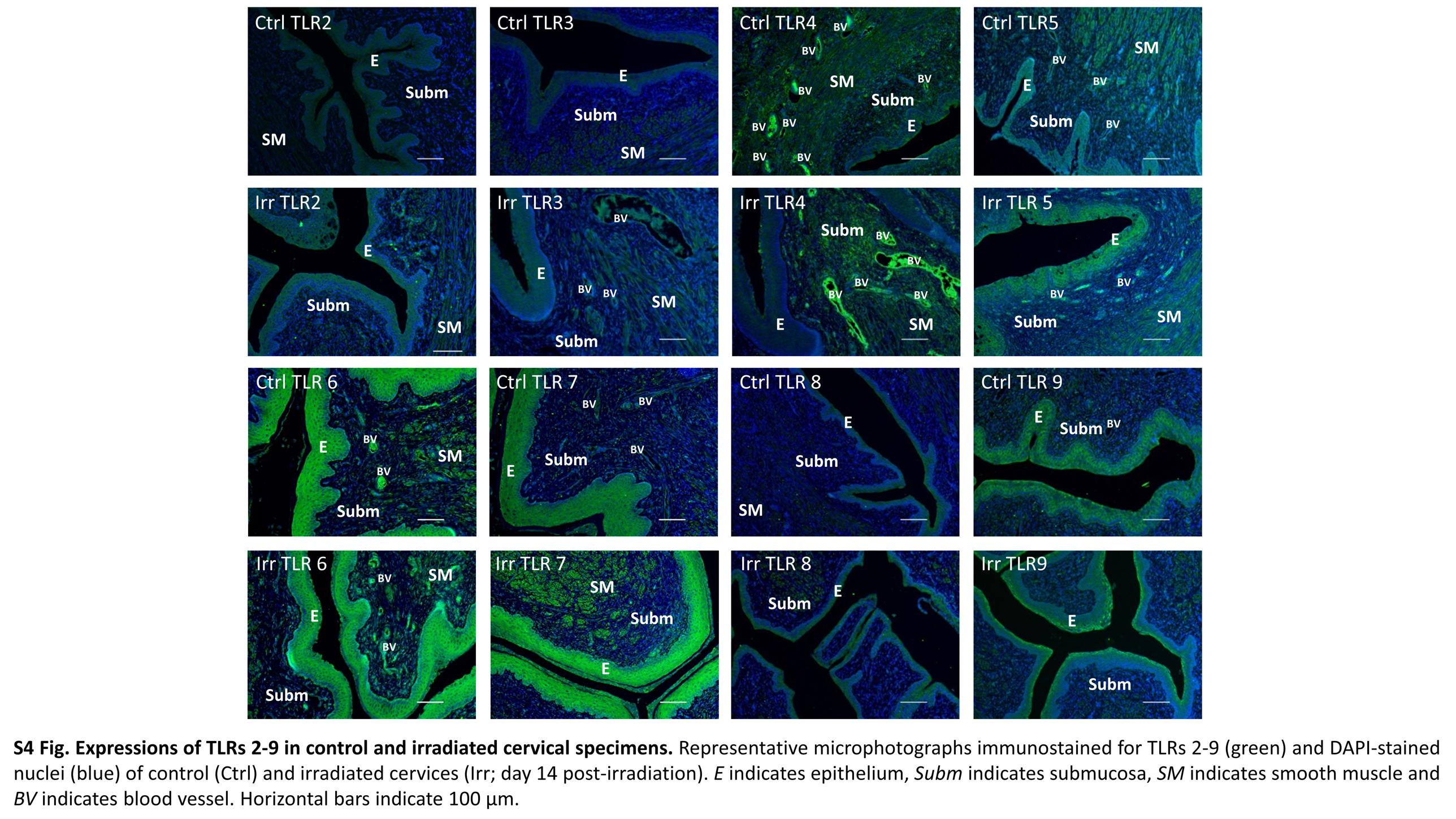

Supplement: S4 Fig — (TIF) [file pone.0215250.s004.tif]
