# Supplementary material for: Radiation induces changes in toll-like receptors of the uterine cervix of the rat
Source: PLoS One. 2019 Apr 18;14(4):e0215250. doi: 10.1371/journal.pone.0215250 (PMC6472742; doi:10.1371/journal.pone.0215250)
Supplement: S1 Table — (DOCX) [file pone.0215250.s005.docx]

**S1 Table. Information on antibodies used for immunohistochemistry and western blot**

|  | **Western blot** | **IHC** | **Manufacturer** | **Reference number** |
| --- | --- | --- | --- | --- |
| mouse anti-α-tubulin | 1:2500 |  | Abcam | T9026 |
| mouse anti-β-actin | 1:2000 |  | Santa Cruz Biotechnology (SCBT) | sc-47778 |
| mouse anti-catalase | 1:500 | 1:100 | SCBT | sc-271803 |
| mouse anti-8-OHdG |  | 1:2000 | Abcam | ab62623 |
| mouse anti-SOD-2 |  | 1:50 | SCBT | sc-137254 |
| mouse anti–TLR9 | 1:500 |  | Sigma Aldrich | WH0054106M3 |
| rabbit anti-CD3e |  | 1:100 | Thermo Fisher Scientific | MA5-14524 |
| rabbit anti-MyD88 | 1:200 | 1:100 | SCBT | sc-11356 |
| rabbit anti-NFκB | 1:1000 |  | Abcam | ab86299 |
| rabbit anti-SOD-1 | 1:50000 | 1:1000 | SCBT | sc-11407 |
| rabbit anti-SOD-2 | 1:500 |  | Abcam | ab13534 |
| rabbit anti-TLR1 | 1:250 |  | SCBT | sc-30000 |
| rabbit anti-TLR1 | 1:500 |  | Abcam | ab180798 |
| rabbit anti-TLR2 | 1:500 | 1:100 | SCBT | sc-10739 |
| rabbit anti-TLR3 | 1:500 | 1:100 | SCBT | sc-28999 |
| rabbit anti-TLR4 | 1:500 | 1:100 | SCBT | sc-30002 |
| rabbit anti-TLR5 | 1:500 | 1:100 | SCBT | sc-30003 |
| rabbit anti-TLR6 | 1:500 | 1:100 | SCBT | sc-30001 |
| rabbit anti-TLR7 | 1:500 | 1:100 | SCBT | sc-30004 |
| rabbit anti-TLR8 | 1:500 | 1:100 | SCBT | sc-25467 |
| rabbit anti-TLR9 |  | 1:100 | SCBT | , sc-25468 |
| rabbit anti-TRIF | 1:1000 | 1:500 | Thermo Fisher Scientific | PA5-23467 |
| Alexa Fluor 488 goat anti-rabbit IgG (H+L) |  | 1:250 | Life Technologies | A11008 |
| goat anti-mouse IgG (H+L)-HRP | 1:4000 |  | Thermo Fisher Scientific | SA248187 |
| goat anti-rabbit IgG-HRP | 1:5000 |  | Abcam | ab6721 |
| Texas red goat anti-mouse IgG (H+L) |  | 1:250 | Life Technologies | T862 |
